# Who uses NHS Direct? Investigating the impact of ethnicity on the uptake of telephone based healthcare

**DOI:** 10.1186/s12939-014-0099-x

**Published:** 2014-11-07

**Authors:** Erica J Cook, Gurch Randhawa, Shirley Large, Andy Guppy, Angel M Chater, Dong Pang

**Affiliations:** Department of Psychology, University of Bedfordshire, Park Square, Luton, UK; Institute for Health Research, University of Bedfordshire, Putteridge Bury, Hitchin Road, Luton, UK; NHS England, Horley, Surrey UK; UCL School of Pharmacy, BMA House, Tavistock Square, London, UK

**Keywords:** NHS Direct, Ethnicity, Remote healthcare, Telephone triage, Healthcare equity, Deprivation

## Abstract

**Introduction:**

NHS Direct, a leading telephone healthcare provider worldwide, provided 24/7 health care advice and information to the public in England and Wales (1998-2014). The fundamental aim of this service was to increase accessibility, however, research has suggested a disparity in the utilisation of this service related to ethnicity. This research presents the first national study to determine how the diverse population in England have engaged with this service.

**Methods:**

NHS Direct call data from the combined months of July, 2010 October, 2010, January 2011 and April, 2011 was analysed (N = 1,342, 245) for all 0845 4647 NHS Direct core service calls in England. Expected usage of NHS Direct was determined for each ethnic group of the population by age and gender and compared by actual usage using Chi-square analysis. A one-way analysis of variance (ANOVA) was used to determine variations of uptake by ethnic group and Index for Multiple Deprivation (IMD) 2010 rank.

**Results:**

Results confirmed that all mixed ethnic groups (White and Black Caribbean, White and Black African, White and Asian) had a higher than expected uptake of NHS Direct which held consistent across all age groups. Lower than expected uptake was found for Black (African/Caribbean) and Asian (Bangladeshi/Indian/Chinese) ethnic group which held consistent by age and gender. For the Pakistani ethnic group usage was higher than expected in adults aged 40 years and older although was lower than expected in younger age groups (0–39).

**Conclusion:**

Findings support previous research suggesting a variation in usage of NHS Direct influenced by ethnicity, which is evidenced on a national level. Further research is now required to examine the underlying barriers that contribute to the ethnic variation in uptake of this service.

## Introduction

NHS Direct, (1998-2014) provided 24/7 nurse-led telephone based health care advice and information [1] to the public in England and Wales. This service supported patients to self-manage symptoms, and where applicable, directed them to the most appropriate form of care. NHS Direct soon became a popular service, handling around 12,000 calls per day, serving nearly 5 million calls per year [2] which was also supported by high levels of patient satisfaction [3,4]. NHS Direct has since been replaced by the new non-emergency NHS 111 telephone based service, However, understanding patterns of NHS Direct usage provides a useful opportunity to learn valuable lessons about telephone healthcare services which can be applied to other similar models of remote healthcare delivery [5-8].

A core aim of NHS Direct was to increase accessibility to healthcare, reducing demand on overstretched NHS services [1,9]. However, research has revealed a wide variation in use by ethnicity, with research suggesting that NHS Direct is underutilised by ethnic minority groups [10,11]. Shah and Cook [10] investigated the socio-economic determinants of A&E and NHS Direct use through the analysis of 2004–5 British General Household Survey. The results suggested that households where the head of the household was not White or born outside the United Kingdom (UK) were significantly less likely to use NHS Direct compared to those of White British ethnic origin [10].

Investigations of ethnic variation have also uncovered an interplay of gender and ethnicity factors which could impact on the uptake of NHS Direct. Bibi, Attwell, Fairhurst & Powell [11] analysed NHS Direct call data (2001–2) against the population structure for the City of Preston, England. For example, white females used the service more than expected, whereby, females from all other ethnic groups used the service less than predicted. However, for males, Black-African, Indian, Pakistani, Bangladeshi, and Asian used NHS Direct more than expected, with particularly high usage found in Indian and Pakistani groups.

Nevertheless, research which has explored the variation of ethnicity have been met by marked criticism. Firstly, NHS Direct utilisation studies have not consistently reported ethnicity [12]. A reason for this may be because most NHS Direct utilisation studies have published studies using call data pre 2003, the point at which NHS Direct began recording ethnicity [13]. Moreover, published studies which have reported ethnicity [10,11,14-17] have relied on small population samples using postal survey methods [14,15,17] and has not reported significant ethnic variation of use. More rigorous studies which have explored the data providing details on uptake by ethnicity are now dated [10,11] and have either been localised studies [11] or relied on non NHS Direct secondary data [10] both of which have lacked national representation.

The presented research therefore endeavours to explore the impact of gender and ethnicity on the uptake of NHS Direct. Through focusing on the national population in England this study aims to provide a more detailed understanding of how the diverse population in England have engaged with a telephone based healthcare service.

## Methods

NHS Direct anonymised call data was collected from the Clinical Assessment System (CAS)^a^ [18] for all 0845 4647 calls made in England for the combined months of July 2010, October 2010, January 2011 and April 2011. There was a total of 1,415,472 calls made, however, all missing cases were excluded from analysis. Ethnicity by gender was available for 85.3% (N = 1,207,046) of all calls with 14.7% (N = 208,426) of cases missing (Males 541,880; Females 665,166). Ethnicity by age was available for 1,206,526 (85.2%) of all calls with 14.8% (208,946) of cases missing, and a total of 1,209,589 cases were available for ethnicity by deprivation with a total of 205,883 cases missing which were subsequently excluded from analysis.

Ethnicity was categorised in line with the census 2001 [19] (see Table 1) as these were the same ethnic groupings that were used by NHS Direct and the Office for National Statistics (ONS) which allowed for the statistical comparison of uptake using population statistics. For age, data was split into five age groups, which included 0–5 years (N = 262,253; 19.8%), 5–19 years (N = 166,771; 12.6%), 20–29 years (N = 260,254; 20.3%), 30–39 years (N = 189,429; 14.3%), 40–59 years (N = 231,480; 17.5%) and 60 years and above (N = 205,333; 15.5%).

**Table 1:** **2001 Census groupings for ethnicity**

**White**	White: British
	White: Irish
	White: Other White
**Mixed**	Mixed: White and Black Caribbean
	Mixed: White and Black African
	Mixed: White and Asian
	Mixed: Other Mixed
**Asian or Asian British**	Asian: Indian
	Asian: Pakistani
	Asian: Bangladeshi
	Other Asian
**Black or Black British**	Black Caribbean
	Black African
	Other Black
**Chinese or other ethnic group**	Chinese
	Other

A Chi-Square goodness of fit test was chosen to compare the NHS Direct categorical demographic call data (ethnicity and ethnicity by age) with general population data. Observed (O) frequencies of callers were compared with expected (E) frequencies. Expected frequencies for uptake by ethnicity were based on the known percentages of each ethnic group derived from ONS current data estimates for population by ethnic group [20] and population by age and ethnic group [21]. To analyse ethnicity by deprivation unit postcodes of included calls were matched to the 2010 Index for Multiple Deprivation (IMD) [22]. A one-way analysis of variance (ANOVA) was conducted to compare ethnic group by 2010 IMD rank^b^. All statistics were completed using IBM SPSS Version 21 [23].

The University of Bedfordshire and NHS Direct approved the study. NHS ethical approval was obtained from the Essex Research Ethics Committee Ref: 10/H0301/29. Ethical approval was provided for the retrospective analysis of anonymized data. Whilst individual written or verbal consent could not be obtained all patients who phone NHS Direct provide on the phone and internet a fair processing message which clearly states that anonymised call records may be used for research purposes. This message provided the caller detailed instructions on how they can withdraw their data from being used. In the case of children it is the parents/guardians responsibility to remove call records if required.

## Results

### Ethnicity and gender

To identify if the ethnicity of both male and female patients was representative of the population in England chi-square goodness of fit statistical analysis was performed. Chi-square analysis confirmed that calls were not representative of the total population in England for ethnicity distribution for neither males (Χ^2^ = 109291.10, df = 15, *p <* .001) or females (Χ^2^ = 215875.52, df = 15, *p <* .001).

For males (Table 2) chi square statistics highlighted that calls for and on behalf of all mixed ethnic groups (White and Black Caribbean, White and Black African, White and Asian, and other) were higher than expected. Mixed White and Asian ethnic sub-group presented the highest over representation with a standardised residual of 305.73. This was followed by mixed (other) and mixed white and Black African with standardised residuals of 73.94 and 54.74 respectively.

**Table 2:** **Chi-square comparison of expected and actual NHS Direct uptake for males compared to the ethnic distribution of the population of England**

**ETHNICITY**	**Observed (O)**	**Expected (E)**	**(O-E)**	**(O-E/√E)**	**Uptake rate**	**Sig**
White: British	441459	451927.9	−0468.9	−15.57	0.98	***
White: Irish	3951	6502.6	−2551.6	−31.64	0.61	***
White: Other	18741	18965.8	−224.8	−1.63	0.99	***
Mixed: White and Black Caribbean	4190	3251.3	938.7	16.46	1.29	***
Mixed: White and Black African	2886	1083.8	1802.2	54.74	2.66	***
Mixed: White and Asian	18623	2709.4	15913.6	305.73	6.87	***
Mixed: Other	5610	2167.5	3442.5	73.94	2.59	***
Asian or Asian British: Indian	12702	14088.9	−1386.9	−11.68	0.90	***
Asian or Asian British: Pakistani	10429	10295.7	133.3	1.31	1.01	NS
Asian or Asian British: Bangladeshi	2450	3793.2	−1343.2	−21.81	0.65	***
Asian or Asian British: Other	4342	3793.2	548.8	8.91	1.14	***
Black or Black British: Caribbean	4367	5960.7	−1593.7	−20.64	0.73	***
Black or Black British: African	4312	8128.2	−3816.2	−42.33	0.53	***
Black or Black British: Other	1478	1083.8	394.2	11.97	1.36	***
Other ethnic groups: Chinese	1181	4335.0	−3154.0	−47.90	0.27	***
Other ethnic group	5159	3793.2	1365.8	22.18	1.36	***

*p* < 0.001***.

However, there was an under representation found on behalf of males who were White (British and Irish), Black (African and Caribbean), Asian (Indian, Bangladeshi and Pakistani) and Chinese. Chi square analysis suggested the lowest representation was found for Chinese (−47.90) African (−42.33) and White Irish (−31.64) and British (−15.57).

For females calls were similarly over represented for all mixed ethnic groups (White and Black Caribbean, White and Black African, White and Asian, and other) (Table 3). Highest over representation was found for mixed White and Asian, White and Black African and mixed other with reported standardised residuals of 445.35, 51.07 and 74.26 respectively.

**Table 3:** **Chi-square comparison of expected and actual NHS Direct uptake for females compared to the ethnic distribution of the population of England**

**ETHNICITY**	**Observed (O)**	**Expected (E)**	**(O-E)**	**(O-E/√E)**	**Uptake rate**	**Sig**
White: British	534087	557409.1	−3322.1	−31.24	0.96	***
White: Irish	5312	7982.0	−2670.0	−29.89	0.67	***
White: Other	25365	23280.8	2084.2	13.66	1.09	***
Mixed: White and Black Caribbean	5619	3991.0	1628.0	25.77	1.41	***
Mixed: White and Black African	3193	1330.3	1862.7	51.07	2.40	***
Mixed: White and Asian	29009	3325.8	25683.2	445.35	8.72	***
Mixed: Other	6491	2660.7	3830.3	74.26	2.44	***
Asian or Asian British: Indian	15003	16629.2	−1626.2	−12.61	0.90	***
Asian or Asian British: Pakistani	11761	11307.8	453.2	4.26	1.04	***
Asian or Asian British: Bangladeshi	2810	4656.2	−1846.2	−27.06	0.60	***
Asian or Asian British: Other	4946	3991.0	955.0	15.12	1.24	***
Black or Black British: Caribbean	6067	7982.0	−1915.0	−21.43	0.76	***
Black or Black British: African	5515	9312.3	−3797.3	−39.35	0.59	***
Black or Black British: Other	2042	1330.3	711.7	19.51	1.53	***
Other ethnic groups: Chinese	1620	5321.3	−3701.3	−50.74	0.30	***
Other ethnic group	6326	4656.2	1669.8	24.47	1.36	***

*p* < 0.001***.

Similarly, as found for males, under representation was found for females who were White (British and Irish), Asian or Asian British (Indian, Pakistani and Bangladeshi), Black or Black British (Caribbean and African) alongside Chinese subgroups.

The greatest under representation was found for Chinese, White British, African and Irish ethnic groups with standardised residuals of −50.74, −31.24, −39.35 and −29.89 respectively.

### Ethnicity and age

To identify if the ethnicity of all users across all age groups was representative of the population in England chi-square goodness of fit statistical analysis was performed. Chi-square analysis confirmed that calls were not representative of the total population in England for patients across all age groups including 0–4 years (Χ^2^ = 19977.48, df = 15, *p <* .001), 5–19 years (Χ^2^ = 30603.76, df = 15, *p <* .001), 20–29 years (Χ^2^ = 70677.71, df = 15, *p <* .001), 30–39 years (Χ^2^ = 64118.65, df = 15, *p <* .001), 40–59 years (Χ^2^ = 49390.81, df = 15, *p <* .001) and patients who were 60 years and older (Χ^2^ = 6659.98, df = 15, *p <* .001).

Observed usage was divided by expected uptake to determine call rate and standardised residuals were calculated to provide significance values (Table 4). For White British uptake rate was lower than expected for children aged 0–4 (0.98) and 5–19 (0.97) years old. However, call rate was either as expected or above for all patients aged 19 years and older. Other White ethnic groups (Irish and White other) showed a variation of uptake by age. For example, higher than expected uptake was found for calls on behalf of White (other) children aged 0–4 years, and White Irish patients aged 0–29 years old. However, lower than expected uptake was revealed in all older age groups. For Mixed ethnic groups who consistently reported a higher than expected uptake across all age groups.

**Table 4:** **Chi-square comparison of expected and actual NHS Direct uptake for age groups compared to the ethnic distribution of the population of England**

**Ethnicity**	**0-4**			**5-19**			**20-29**			**30-39**			**40-59**			**60+**		
	**N**	**Uptake rate**	**Sig**	**N**	**Uptake rate**	**Sig**	**N**	**Uptake rate**	**Sig**	**N**	**Uptake rate**	**Sig**	**N**	**Uptake rate**	**Sig**	**N**	**Uptake rate**	**Sig**
White: British	185267	0.98	***	121137	0.97	***	188761	1.03	***	130402	1.00	NS	176923	1.00	*NS*	168110	1.01	*
White: Irish	889	1.02	*NS*	844	1.79	***	1936	1.36	***	1444	0.88	***	1724	0.65	***	2385	0.64	***
White: Other	13694	2.40	***	3819	0.98	*NS*	10170	0.73	***	9476	0.86	***	5703	0.80	***	3528	0.95	***
Mixed: White and Black Caribbean	3867	1.08	***	1768	0.83	***	2287	1.15	***	1148	1.62	***	892	1.86	***	226	1.77	***
Mixed: White and Black African	2195	1.15	***	1189	1.81	***	1478	2.00	***	662	1.53	***	502	1.72	***	99	1.36	***
Mixed: White and Asian	9070	1.58	***	9026	4.74	***	14467	6.00	***	9569	8.14	***	6287	9.15	***	1217	5.55	***
Mixed: Other	5501	1.63	***	2154	1.83	***	2111	1.43	***	1185	1.52	***	895	1.72	***	242	1.33	***
Asian or Asian British: Indian	5937	0.89	***	3070	0.80	***	6392	0.61	***	5646	0.82	***	4343	0.80	***	2285	0.94	**
Asian or Asian British: Pakistani	5268	0.66	***	3154	0.76	***	5885	0.80	***	3899	0.86	***	2794	1.04	**	1185	1.18	***
Asian or Asian British: Bangladeshi	1384	0.45	***	775	0.45	***	1471	0.49	***	887	0.49	***	469	0.52	***	271	0.82	***
Asian or Asian British: Other	2149	0.90	***	1121	1.04	NS	2165	0.77	***	1836	0.97	*NS*	1400	1.02	*NS*	598	1.17	***
Black or Black British: Caribbean	1702	0.72	***	1451	0.85	***	2447	0.81	***	1571	0.79	***	2360	0.76	***	894	0.56	***
Black or Black British: African	2324	0.45	***	1276	0.46	***	2504	0.44	***	2062	0.52	***	1390	0.48	***	265	0.45	***
Black or Black British: Other	774	0.65	***	547	0.94	NS	876	1.08	*	541	1.30	***	625	1.50	***	147	1.61	***
Other ethnic groups: Chinese	585	0.33	***	261	0.30	***	799	0.18	***	605	0.23	***	365	0.24	***	172	0.35	***
Other ethnic group	2355	1.71	***	1182	1.27	***	2838	0.75	***	2259	0.92	***	1744	1.12	***	972	2.31	***

*p* < 0.05* *p* < 0.01** *p* < 0.001*** *NS* > .05.

For Asian (Bangladeshi and Indian) ethnic groups there was a lower than expected uptake rate across all age groups (p < .001). Highest uptake rate was found for both Bangladeshi and Indian patients who were 60 years and older, with an uptake rate of 0.82 and 0.94 respectively (p < .001). However, lowest uptake rate was found in children aged four years and younger (0.45, p < .001) for Bangladeshi patients and for patients aged 20–29 (0.61, p < .001) for Indian patients.

A pattern emerged for Pakistani patients, whereby the older the patient the higher the uptake rate. For example, uptake rate for children aged 0–4 years was 0.66, which subsequently increased for patients aged 5–19 years (0.76), 20–29 years (0.80), and 30–39 years (0.86). The pattern continued with over representation found in patients aged 40–59 years (1.04) and 60 years and older (1.18).

For Black Caribbean patients lower than expected uptake rate was found across all ages. The uptake rate ranged from 0.72-0.76 (p < .001) in patients aged 0–59 years with the lowest uptake rate found in patients aged 60 years and older (0.56, p < .001). Black Africans highlighted lower than expected uptake rate (0.45-0.52) consistently across all age groups (p < .001) showing limited variation of uptake by age group. Finally, Chinese patients highlighted the lowest uptake compared to all ethnic groups, which remained consistent across all age groups (p < .001). The lowest uptake rate was found for Chinese patients aged 20–29 years (0.18; p < .001) with all other age groups showing an uptake rate range of 0.24-0.35 (p < .001).

### Ethnicity and deprivation

**Figure 1:**
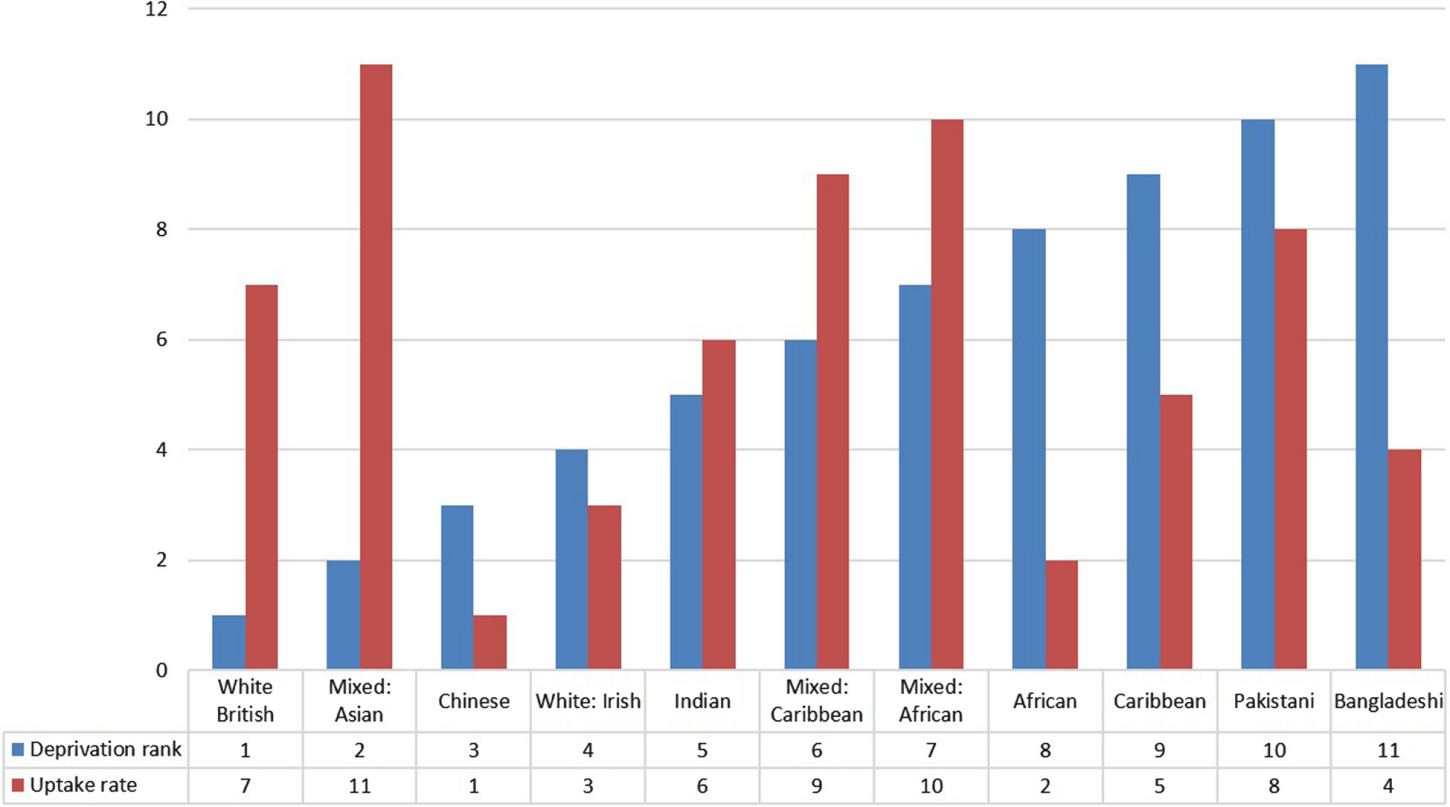
**Deprivation and uptake rank across all ethnic groups.**

A one-way ANOVA was conducted to determine mean difference of 2010 IMD rank by ethnicity. The analysis was significant (*F*(15, 1 209,573) =2416.60 p < .001), Bonferroni post-hoc tests was used to look at between group differences. Figure 1 presents the ranked order of deprivation and ranked order of uptake by ethnic group, whereby a deprivation rank of 1 is the lowest deprived rank across all ethnic groups and the uptake rank of 1 is lowest uptake rate rank across all ethnic groups.

Bangladeshi (M = 5211; SD = 7432.69) Pakistani (M = 8188.58; SD = 7666.72), African (M = 9421.21; SD = 78.27) and Caribbean (M = 9077.79; SD = 7007.32) ethnic groups had significantly higher levels of deprivation compared to all other ethnic groups (p < .001). All of these groups (except Pakistani for older age groups) presented a lower than expected uptake rate.

However, in contrast Chinese (M = 15081.26; SD = 9272.80) and Indian ethnic groups (M = 13380.35; SD = 8703.52) who had lower than expected uptake rate of NHS Direct had lower levels of deprivation. White British (M = 15789.55 SD = 9300.28) and Mixed White and Asian (M = 15627.60; SD = 9352.58) who represent expected or higher than expected uptake of NHS Direct across all age groups had the lowest levels of deprivation compared to all ethnic groups (p < .001).

## Discussion

### Main findings of this study

The research presented here highlights that across a national representation of NHS Direct users there is a variation to uptake by ethnicity which holds consistent by gender. The findings revealed that there was higher representation of uptake for all mixed ethnic groups (White and Black Caribbean, White and Black African, White and Asian, and other) for both males and females. This was particularly evident for the mixed White and Asian which accounted for the greatest over representation across all ethnic groups.

The White British population in England have been consistently shown to be the highest users of NHS Direct [10,11]. However, this study revealed that this ethnic group had lower representation than expected. This finding may an artifact of age, deprivation and geographic factors which have been found to impact on uptake of this service. For example, the older population have been shown to be the lowest users and yet represent a large section of the national population [17]. Research has also shown that there is a variation of usage of NHS Direct by deprivation and geographic location [12,24,25]. Low representation was also found for Asian and Black ethnic minority groups, with lowest observed usage found for Chinese and Black African male and female ethnic sub-groups. This finding is supported by previous research which has also highlighted lower observed uptake of NHS Direct in ethnic minority groups that were of Asian and Black ethnic origin [10].

For all White ethnic groups there was a variation of uptake by age. For example, White British uptake rate was lower than expected for calls relating to young children (0–4) with expected or higher than expected usage found for all other age groups. Conversely, analysis revealed that Irish and White Other ethnic groups showed highest uptake in calls relating to young children (0–4) with lower than expected uptake was revealed in all older age groups.

The findings also revealed that the lowest users of NHS Direct (Chinese, Bangladeshi, Black African and Caribbean) had low uptake rates across all age groups. However, interestingly, whilst the Pakistani ethnic group showed higher than expected uptake across both genders uptake varied by age group, whereby increased age was associated with increased uptake. Whilst previous research has suggested that uptake of NHS Direct is lower in older age groups the impact of ethnicity has not been evaluated, therefore this has emerged as a positive public health finding [24,26]. Moreover, this finding provides a useful avenue for further research to understand why uptake is lower than expected in younger age groups (0–39).

For deprivation it was found that the lowest users of NHS Direct had the highest levels of deprivation and the highest users of NHS Direct had the lowest levels of deprivation (White British and Mixed White and Asian). However, this was not the case for Indian and Chinese ethnic groups who despite having lower levels of deprivation they demonstrated lower than expected uptake rate which held consistent for age and gender. Whilst it is know that deprivation has impacted on NHS Direct uptake [24,25] the current research highlights variation of uptake by levels of deprivation related to ethnicity.

### What is already known on this topic

Previous research has highlighted that individual’s from minority ethnic groups within the UK have experienced poorer health and barriers in accessing certain health care services [27,28]. Furthermore, different levels of use of healthcare services are evident, which has become an important priority for governments worldwide to overcome [29]. However, there has been limited research which has explored ethnic distribution on the uptake of NHS Direct. Previous studies have highlighted that this service is under-used by certain ethnic groups with this difference depending on gender [11]. For example, females from all ethnic groups combined have been associated with lower uptake. Conversely, males who are Black African, Indian, Pakistani, Bangladeshi and Asian groups have been associated with higher uptake [11].

### What this study adds

This research attempts to engage with current debate in how individuals engage with telephone based healthcare, and highlights how a relatively new innovative service has engaged ethnic sub-groups of the population since its inception. This is the first study that has adopted a national sample to examine how ethnicity by age, gender and deprivation interact to explain uptake of NHS Direct, a national telephone based healthcare service.

In contrast to previous research, this study has uncovered that mixed ethnic groups are engaging with this service more than expected, with this finding consistent across both genders. This study has also provided useful information about how uptake across the diverse population in England is influenced by age. Whilst there are ethnic groups who are not engaging with this service across all age groups this finding is not consistent for all ethnic groups. For example, whilst the Pakistani ethnic group have shown higher than expected uptake this is not consistent across age groups with lower than expected uptake found for younger age groups. Therefore, it remains an important priority to determine accessibility issues for minority ethnic groups. Particularly for South Asian (Indian/Bangladeshi/Pakistani) and Black (African/Caribbean) ethnic sub-groups as they continue to represent the highest users of both primary healthcare services for both in-hours and out-of-hours healthcare [30-32].

The differences in uptake in previous studies that have looked at healthcare utilisation have related ethnic variations to differences in health-seeking behaviour, or difficulties in accessing high-quality primary care services [32]. The fact that NHS Direct provided an interpreter service through ‘language line’ may not be well recognised and it is unclear how this was promoted. Therefore, future research could explore the knowledge of this in communities who are not native English speakers. Moreover, further exploration is needed to understand the fundamental barriers and facilitators which impact on utilisation of telephone based healthcare and essentially the factors that may impact on the uptake of NHS Direct which will be applicable to the new ‘111’ service.

### Limitations of this study

There are some limitations that should be considered. There was a significant proportion of data missing which was excluded from analysis. For example, there was a total of 208,426 cases missing for ethnicity and gender and 208,946 cases missing for ethnicity and age. Missing data represents a key challenge to the analysis of secondary data [33] and as such a detailed overview of data missing by ethnicity and gender was made before deciding on exclusion. After investigation the main reason for the missing call data which explained 80.5% (N = 167,782) of all missing cases for gender and ethnicity was due to ‘quick calls’. These are calls which are mainly dealt with by the health advisor whereby no further action is needed. After excluding these calls there was a total of 40,644 calls missing which only contributed to 3.0% of the total data. Missing cases can also be attributed urgent or emergency calls, or periods of very high demand when question deliberately not asked. After a detailed validation of the remaining missing cases they were not shown to be systematically different from those with complete data. Therefore it was concluded that these calls were Missing Completely at Random (MCAR) and could be an artefact of data entry error and consistency in data recording practices across the NHS Direct call centres. Nevertheless, there remained a large number of calls included in analysis which represented over 85% of all calls made across the four months. Therefore the excluded calls was not felt to impact on the statistical interpretation of analysis.

The way ethnicity is measured has changed from the 2001 census [19] to the 2011 census [34]. The latest census now includes ‘Gypsy or Irish Traveller’ with a write in option for ‘other’ categories. Whilst it would have been preferable to use more accurate and current population statistics the first data extraction was before the 2011 census so the data was restricted to the 2001 census ethnicity groupings. Whilst the current research has explored ethnicity by gender, ethnicity by age and ethnicity by deprivation it has not explored the interaction of ethnicity by age by deprivation. This would provide a useful avenue for future research to explore if older people from certain ethnic backgrounds behave differently to second and third generations who are younger.

## Conclusions

This research has provided a fuller understanding of how the population in England engage with NHS Direct, highlighting that there are certain sections of the population who were found to be low users compared to other sections of the population. Importantly, this research has uncovered that that both male and female Pakistani’s alongside mixed ethnic groups have shown higher uptake than previous research, demonstrating a positive public health finding. This has important implications for telephone based healthcare both nationally, including the future national health applications, such as the new NHS 111 service [35] and internationally where telephone based health care systems have become the model of international healthcare e.g. Ontario in Canada [5,6], and Health Direct in Australia [7]. Research is now needed to explore ethnic sub-groups of the population that are considered low users of NHS Direct to explore perceptions and attitudes towards telephone based healthcare to determine barriers and facilitators of uptake.

## Endnotes

^a^CAS is an evidence-based algorithm tool used by NHS Direct nurses to assist the triage of patients.

^b^All 32,482 lower super output areas in the UK are put into a rank order based on their 2010 IMD score. A rank of 1 is the most deprived.

## Abbreviations

ANOVA: Analysis of variance
CAS: Clinical Assessment System
IMD: Index for Multiple Deprivation
MCAR: Missing Completely At Random
UK: United Kingdom
